# Self-reported knowledge, attitudes and concerns about zoonoses among general practitioners in the Netherlands

**DOI:** 10.1016/j.onehlt.2025.101121

**Published:** 2025-06-27

**Authors:** F. Dusseldorp, C.F.J. Vlaanderen, S. Feenstra-Gols, J.W.B. van der Giessen, L. Mughini-Gras

**Affiliations:** a Centre for Infectious Disease Control, National Institute for Public Health and the Environment (RIVM), Bilthoven, the Netherlands; b Institute for Risk Assessment Sciences (IRAS), Faculty of Veterinary Medicine, Utrecht University, Utrecht, the Netherlands

**Keywords:** Zoonosis literacy, Health literacy, One health, General practitioners

## Abstract

**Introduction:**

Adequate zoonosis literacy among general practitioners (GPs) is essential for signaling and managing zoonotic outbreaks and potential emerging infectious diseases (EIDs). This study aimed to assess Dutch GP's knowledge, attitudes and concerns about zoonoses and their impact on GPs' self-reported confidence in diagnosing and screening for zoonotic risk factors in daily practice.

**Method:**

Data was collected in 2023 using an online questionnaire directed at GPs working in clinical practice in the Netherlands. A multivariable logistic regression analysis was applied to assess whether there were significant associations of socio-demographic factors and other characteristics of GPs with their self-reported confidence.

**Results:**

Of the 332 participating GPs, 12.1 % reported feeling confident in diagnosing and managing zoonoses, and 44.1 % felt insecure about this. The vast majority (80.4 %) indicated that they rarely or never discuss potential risks and prevention strategies for zoonoses in relevant patients. GPs were relatively less likely to feel insecure about zoonoses if they had more years of practice, a better knowledge about infectious disease notification laws, reported notifiable diseases to municipal health services, and diagnosed zoonoses more than once a year. In patients with neurological diseases, fever, infectious respiratory diseases, and skin infections, 44 %, 42 %, 32 % and 9 % of GPs reported never asking about contact with animals or exposure to nature, respectively.

**Conclusion:**

This study revealed that awareness about zoonoses and confidence in diagnosing them are generally low among Dutch GPs. A low response rate and the self-reported data may have led to an overestimation of their confidence. Therefore, additional training and targeted information would be beneficial to enhance awareness**.**

## Highlights

• Most Dutch GPs report having received insufficient training about zoonoses.
• Most Dutch GPs report never discussing zoonosis risks and prevention.
• Over 40 % of Dutch GPs feel unsure about diagnosing and managing zoonoses.
• Dutch GPs seldom inquire about zoonotic exposure in various clinical scenarios.

## 1 Introduction

Emerging infectious diseases (EID) are defined as infectious diseases that have either newly appeared in a population or have existed previously, but are rapidly increasing in incidence or geographic spread [1]. Most (75 %) EIDs are zoonotic, meaning they originated from a vertebrate animal host and crossed the species barrier to infect humans [2]. When humans can infect one another, it can lead to larger outbreaks or even pandemics.

Several factors that facilitate closer contact between humans and animals favor the spread of zoonotic pathogens, thereby driving the spread of EIDs [3,4].

The COVID-19 pandemic highlighted the severe impact of EIDs and urged nations to enhance response to zoonotic threats. Following recommendations from the expert report by Bekedam et al., the Dutch government developed a national action plan to strengthen policies aimed at combating zoonoses, with “zoonosis literacy’ as key theme [5,6]. This parallels the concept of health literacy, as it emphasizes the ability of individuals to obtain, process, and understand basic health information and services [7], specifically about zoonoses. This is not only relevant for the public, but also for professionals, such as general practitioners (GPs), to improve health crises preparedness [1].

GPs play a vital role in frontline healthcare and signaling of EIDs, including zoonotic infections, in humans. It is crucial that GPs are able to timely recognize zoonoses and know when and how to notify them to public health authorities, including reporting unknown pathogens with epidemic potential. This enables timely action, such as public health interventions and resource allocation, to help mitigate the spread of the disease.

GPs tend to discuss the potential risk of zoonotic diseases - or strategies to prevent them - less often than veterinarians, and show generally less confidence in diagnosing zoonoses than veterinarians [8,9]. Known factors associated with diminished confidence in diagnosing and managing zoonoses include limited training in zoonoses and infectious disease epidemiology [8]. Despite the significance of zoonoses in the Netherlands and the recognition of zoonosis literacy as an important issue, there is still limited information about the knowledge, attitudes and concerns regarding zoonoses among GPs. It also remains unclear whether Dutch GPs are sufficiently aware about how to report signals of infectious diseases to national notification systems [6].

The aim of this study was to assess knowledge, attitudes and concerns about zoonoses among Dutch GPs, as well as to determine how these may influence GPs' self-reported confidence in diagnosing and screening for zoonotic risk factors in daily practice. This information is expected to provide insights whether there is need to improve zoonosis literacy among Dutch GPs.

## 2 Materials and methods

### 2.1 Study design

This study was based on a cross-sectional design using an online questionnaire directed at GPs working in clinical practice in the Netherlands. With a population of 4640 GPs, a sample size of 302 participants would be sufficient to measure binary variables with an expected prevalence of 30 %, achieving a desired precision of 0.05 and a confidence level of 95 %.

The questionnaire was developed in consultation with experts in the human and veterinary fields, to assess GP's confidence of zoonoses and whether they consider a zoonosis in certain clinical situations. In May 2023, it was distributed to 4640 licensed GPs in the Netherlands, listed in the AGB-register. After piloting with five GPs to assess accuracy and comprehensibility, and to determine the average time to respond, only small linguistic changes were needed. The self-administered online questionnaire included 19 questions about GPs' self-confidence, experience and behavior regarding zoonotic diseases (see Supplement 1 for the full survey). The questionnaire also gathered demographic data, including the year of birth, year of graduation as medical doctor, and the location of the GP practice: province and type of environment within the patients' catchment area (city, village or rural).

### 2.2 Representativeness of participants

The demographic data of the participating GPs was compared to the Dutch GP registration data of the Netherlands Institute for Healthcare Services Research (Nivel) of 2020–2022 to assess the sample's representativness relative to the National GP population. Nivel data tracks the number of active GPs and specific demographic detail of the profession.

### 2.3 Statistical analysis

Data collected with the survey were reported descriptively using absolute numbers and proportions, mean (standard deviation [SD]) or median (interquartile range [IQR]) values, as appropriate. To compare age between participants and Nivel's GP data, the one-sample two-tailed *t*-test was used, while differences in gender and province of practice (categorical variables) were tested using Pearson's chi-square test.

Logistic regression was used to identify variables significantly associated with the level of GPs' self-reported confidence in diagnosing and managing zoonoses. Questions about self-reported confidence were scored on a five-point Likert scale (very insecure, insecure, neutral, secure or very secure) and then dichotomized as “not insecure” (neutral, secure and very secure), coded as 0, and “insecure” (insecure or very insecure), coded as 1, for regression analysis. We asked GPs whether they usually ask patients about contact with animals and/or exposure to nature, as a proxy for screening for potential risk factors for zoonoses in seven hypothetical clinical situations (sickness after travelling, infectious disease in pregnancy, disease in people with an occupational risk for zoonoses, infectious respiratory diseases, neurological diseases, skin infection and fever). For the hypothetical situations of patients with fever, infectious respiratory diseases, neurological diseases and skin infections, we used logistic regression to identify variables associated with GPs who screen for potential risk factors for zoonoses in these patients. The question was answered on a four-point Likert scale (never, sometimes, in most cases, always) and was dichotomized for regression as never/sometimes (0) and most/always (1). Odds ratios (OR) and 95 % confidence intervals (95 %CI) were estimated using either ‘insecure’ or ‘never/sometimes’ as the reference groups.

Gender was coded as ‘no male’ (0) and ‘male’ (1), and location of practice as ‘being in the conurbation of the Netherlands’ (provinces Flevoland, South-Holland, North-Holland and Utrecht) No (0) or Yes (1) [10]. Education about zoonoses was coded as non-existent to insufficient (0) or sufficient to good (1). Confidence as a GP overall and in diagnosing infectious diseases in particular was coded in a similar way as the aforementioned outcome variable confidence in diagnosing and managing zoonoses. Five-point Likert-scale data concerning zoonoses and knowledge about notification law for infectious diseases were collapsed into 3 outcomes (1 + 2, 3 and 4 + 5). Due to low frequencies, the five point Likert-scale data concerning those about talking about risks and preventive measures for zoonoses with relevant patients, and the number of contacts with public authorities about notifiable diseases in the past, were collapsed into 2 outcomes (1 + 2 vs. 3 + 4 + 5, and 1 vs. 2 + 3 + 4 + 5, respectively). The number of zoonotic diagnoses per year was recoded as less than once (0) or ‘once or more’ (1).

Variables with *p* < 0.10 in univariable logistic regression were considered the multivariable logistic model, with year of graduation and gender always included. A backward stepwise variable selection procedure was then applied to retain in the multivariable models only those variables significantly associated with the outcome at *p* < 0.05. Multicollinearity was assessed prior to multivariable analysis using the variance inflation factor (VIF). All analyses were conducted in Stata (version 15.1, StataCorp, College Station, Tx,).

## 3 Results

### 3.1 Sample description

In total, 332 participants out of the 4640 invited completed the survey (response rate = 7.15 %). One participant was a GP in training and was therefore excluded. Median age of participants was 45 (range 32–69) years. There were relatively more female GPs (64.35 %) and most participants worked in the most densely populated provinces of the country: Zuid-Holland and Noord-Holland. Participants did not differ significantly from the overall GP population in the Netherlands in terms of mean age, gender and province of GP practice (Table 1).

**Table 1:** Participant characteristics of this study compared to the overall GP population in the Netherlands (Nivel data).

Variable	Participants (%)	Nivel data (%)	*p*-value
Age in years (mean)	47.17	47	0.74
Gender			0.31
Female	64.35	60.6	
Male	35.05	39.4	
Other	0.6	0	
Province of GP practice⁎			0.16
Friesland	3.3	3.8	
Groningen	2.7	3.4	
Drenthe	3.3	2.9	
Overijssel	4.8	6.5	
Gelderland	11.5	12.7	
Flevoland	1.2	2.4	
Noord-Brabant	15.4	13.8	
Limburg	6.3	6.8	
Zeeland	1.8	2.0	
Zuid-Holland	22.7	20.2	
Noord-Holland	21.8	17.0	
Utrecht	5.1	8.7	

⁎ GPs report to Nivel all provinces in which they work, but in our data the participants reported only the province in which they worked the most.

#### 3.1.1 Infectious diseases in general

Most participants (66.1 %) felt secure in diagnosing and managing infectious diseases, and 30.2 % indicated that they were well to very well informed about their legal obligations to report notifiable infectious diseases to public health authorities. The majority (82.8 %) of GPs reported having consulted public health authorities about infectious diseases.

#### 3.1.2 Zoonoses

Of all participants, 13.9 % were very to extremely worried about recent developments regarding zoonotic diseases and their impact on public health, and 38.7 % were fairly worried. The majority (80.7 %) stated that zoonoses were not to insufficiently addressed during their GP training. Moreover, only 12.1 % of GPs stated that they felt secure in diagnosing and managing zoonoses, and 44.1 % felt insecure about this. The vast majority indicated that they rarely to never (80.4 %) discussed potential risks or prevention strategies for zoonoses in relevant patients. More than half of GPs (59.5 %) reported diagnosing zoonoses less than once a year.

### 3.2 Factors associated with confidence in diagnosing and managing zoonoses

Due to high correlation between year of graduation and GP age, we excluded age from the multivariate regression models, as year of graduation was considered the most accurate indicator for experience as GP. While adjusting for other variables in the model and after performing backward variable selection, the following variables were significantly associated with decreased odds of feeling insecure about diagnosing and managing zoonoses (Table 2): graduation before 2003 vs. after 2013, (adjusted OR [aOR] 0.37, 95 % CI 0.18–0.77), being fairly (aOR 0.33 95 % CI 0.16–0.68) and good to very good (aOR 0.14 95 % CI 0.025–0.78) informed about infectious disease notification laws (vs. being not to only little informed about it), having reported one or more notifiable diseases in the last year to municipal health services vs. never have done so (aOR 0.45 95 % CI 0.25–0.81), and usually diagnosing zoonoses once or more than once a year vs. less than once a year (0.41 aOR 95 % CI 0.23–0.73).

**Table 2:** Final results from the multivariable logistic regression analysis of factors associated with feeling insecure about diagnosing and managing zoonoses.

	Insecure about zoonosis	Not insecure about zoonosis	p-value	Adjusted OR⁎ (95 % CI)	p-value
GP gender (male) (%)			0.034		
No male	104 (71.2)	111 (60.0)			
Male	42 (28.8)	74 (40)			
Graduation year as a GP (%)					
After 2013	57 (39.0)	56 (30.3)			
2003–2013	62 (42.5)	68 (36.8)	0.669		
Before 2003	27 (18.5)	61 (33.0)	0.005	0.37 (0.18–0.77)	0.008
Living in the conurbation of the Netherlands			0.4921		
No	75 (51.4)	88 (47.6)			
Yes	71 (46.6)	97 (52.4)			
Studied or worked in a foreign country			0.284		
No	101 (70.6)	117 (65.0)			
Yes	42 (29.4)	63 (53.0)			
Settlement of the GP practice					
Large city	42 (29.2)	51 (27.7)			
Small city	44 (30.6)	52 (28.3)	0.926		
Village/rural	58 (40.3)	81 (44.0)	0.605		
Feeling insecure as a GP in general			0.244		
No	143 (98.0)	184 (99.5)			
Yes	3 (2.1)	1 (0.5)			
Feeling insecure about diagnosing infectious diseases			0.005		
No	129 (88.4)	179 (96.7)			
Yes	17 (11.6)	6 (3.2)			
Informed about infectious disease notification law					
No to a little	35 (29.4)	14 (11.5)			
Fairly	82 (68.9)	99 (81.2)	0.002	0.33 (0.16–0.68)	0.003
Well to very well	2 (1.7)	9 (7.4)	0.004	0.14 (0.02–0.78)	0.025
Self-reported number of reports of notifiable infectious diseases			0.011		
Zero	102 (69.9)	104 (56.2)			
Once or more than once a year	44 (30.1)	81 (43.4)		0.45 (0.25–0.81)	0.008
Had contact with infectious disease teams of public health authorities in the past year			0.524		
No	23 (16.1)	29 (15.7)			
Yes	120 (83.9)	154 (83.2)			
Amount of education regarding zoonoses during their GP training			0.002		
Absent to insufficient	129 (88.4)	138 (74.6)			
Sufficient to abundant	17 (11.6)	47 (25.4)			
Worries about impact of zoonotic diseases on public health					
No to a little	74 (50.7)	83 (44.9)			
Fairly	54 (37.0)	74 (40.0)	0.404		
Worried to very worried	18 (12.3)	28 (15.1)	0.339		
Self-reported number of zoonotic diagnoses in the past year			0.000		0.002
Less than one	105 (71.9)	92 (49.7)			
Once or more than once	41 (28.1)	93 (50.3)		0.41 (0.23–0.73)	
Ever advised a patient to consult a vet			0.019		
No	97 (69.8)	103 (56.9)			
Yes	42 (30.2)	78 (43.1)			
Has had a patient with a referral from a vet			0.107		
No	130 (89.1)	153 (82.7)			
Yes	19 (11.0)	32 (17.3)			
Discusses risks and preventive measures regarding zoonoses with relevant patients			0.017		
Never to rarely	126 (86.3)	140 (75.7)			
Sometimes to always	20 (13.7)	45 (24.3)			

OR = odds ratio. 95 % CI = 95 % confidence interval.

⁎ After performing backwards selection.

### 3.3 Screening for risk factors for zoonoses in specific patients

**Fig. 1:**
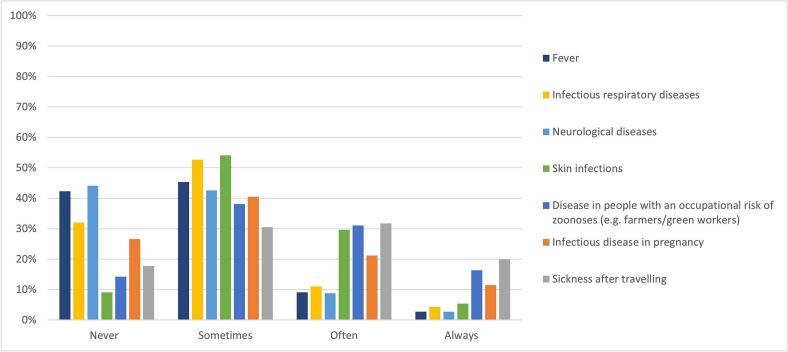
Percentages of GPs who never/sometimes/often or always ask about contact with animals or exposure to natural environments in several clinical situations.

In patients with neurological diseases, fever, infectious respiratory diseases, and skin infections, 44 %, 42 %, 32 % and 9 % of GPs reported never asking about contact with animals or exposure to nature, respectively (Fig. 1). Among patients presenting with fever, 12 % of the GPs report often or always asking about contact with animals or exposure to nature, while 45 % report making such inquiries sometimes. For patients with infectious respiratory diseases, 15 % of the GPs often or always asking about contact with animals or exposure to nature, and 53 % reports doing so sometimes. In patients who are sick after travelling, who have an occupational risk of zoonoses, and have an infectious disease in pregnancy, 20 %, 16 % and 12 % of GPs reported always asking about contact with animals or exposure to nature, respectively.

#### 3.3.1 Patients with fever

Being generally insecure (vs. not insecure) as GP (aOR 15.25 95 % CI 1.59–146.4) and sometimes to often (vs. rarely to never) discuss risks and preventive measures regarding zoonoses with relevant patients (aOR 3.00 95 % CI (1.44–6.26)), were all significantly associated with higher odds of asking about exposure to nature or contact with animals in patients with fever. Conversely, being insecure about zoonoses was significantly associated with lower odds (aOR 0.29 95 % CI 0.12–0.68).

#### 3.3.2 Patients with neurological diseases

Having been in contact with public health authorities about notifiable disease once or more than once in the past year vs. never having done so (aOR 2.11 95 % CI 1.03–4.35), having ever (vs. never) advised a patient to consult a veterinarian to have their animals treated for possible zoonoses (aOR 2.28 95 % CI 1.1–4.71), and sometimes to often discuss risks and preventive measures regarding zoonoses with relevant patients vs. rarely to never doing so (aOR 2.31 95 % CI 1.07–4.99), were all significantly associated with higher odds of asking about exposure to nature or contact with animals in patients with neurological disease. Conversely, living in the conurbation of the Netherlands was significantly associated with lower odds (aOR 0.45 95 % CI 0.21–0.95).

#### 3.3.3 Patients with infectious respiratory diseases

Having ever (vs. never) advised a patient to consult a veterinarian (aOR 2.45 95 % CI 1.29–4.64) and sometimes to often discuss risks and preventive measures regarding zoonoses with relevant patients vs. rarely to never doing so (aOR 2.11 95 % CI 1.05–4.24) were both associated with higher odds of asking about risk factors for zoonoses in patients with infectious respiratory disease.

#### 3.3.4 Patients with skin infections

Being generally insecure (vs. not insecure) concerning zoonoses (aOR 0.44 95 % CI 0.25–0.76) and living (vs. not living) in the conurbation of the Netherlands (aOR 0.54 95 % CI 0.32–0.91) were associated with lower odds of asking about risk factors for zoonoses in patients with skin infections. Having ever (vs. never) advised a patient to consult a veterinarian to have their animals treated with for possible zoonoses (aOR 2.03 95 % CI 1.20–3.44), discussing sometimes to often risks and preventive measures regarding zoonoses with relevant patients vs. rarely to never doing so (aOR 1.85 95 % CI 1.25–2.74), and reporting to usually diagnosing zoonoses once or more than once a year vs. doing it zero times a year (aOR 1.86 95 % CI 1.08–3.20) were all significantly associated with higher odds of asking about exposure to nature or contact with animals in patients with neurological disease.

## 4 Discussion

This study explored self-reported confidence regarding zoonoses and associated factors among Dutch GPs, as well as when GPs ask for potential exposures to zoonotic agents in the anamnesis. A considerable number of GPs in this study reported feeling insecure about zoonoses and the vast majority felt that zoonoses were insufficiently addressed during their GP training. Most GPs reported that they rarely, if ever, discuss potential risks and preventive measures related to zoonoses, and the majority indicated that they diagnose fewer than one zoonosis per year.

Numerous infectious diseases, including foodborne infections, are zoonoses. Therefore, it seems unrealistic that GPs diagnose so few zoonoses each year. This may reflect low awareness among GPs, as seen in Australia studies linking low awareness of zoonotic diseases among GPs to insufficient training in One Health [8,11]. The One Health concept may encourage GPs to consider the environment and animals that patients interact with, when assessing their health situation. However, the concept of One Health, zoonoses, and engagement with public health services, are no emphasized themes in the standard national GP training program in the Netherlands [12]. Unlike veterinary education, which is focused to a large extent on animal infectious diseases, with emphasis on zoonoses. Accordingly, the vast majority of GPs in our study felt their medical training insufficiently addressed zoonoses, and consequently a significant part of GPs felt insecure about handling zoonoses in their practice. Integrating One Health modules into GP residency programs might improve confidence among GPs in diagnosing and managing zoonosis.

GPs who considered themselves well-informed about the infectious disease notification law and who reported to contact public health authorities once or more often in the past year had lower odds of feeling insecure about diagnosing and managing zoonoses. This suggests that strengthening interaction between public health services and GPs is likely to improve awareness about zoonoses among GPs and therefore improve signaling of relevant cases.

Indicated and care-related prevention are seen as important tasks for primary care according to GPs in the Netherlands [13]. Discussing the risks and preventive measures regarding zoonoses is a form of indicated prevention, as it involves prevention of diseases based on risk factors in individuals. However, this study showed that the vast majority of respondents stated that they rarely or never (80.4 %) discuss potential risks or strategies to prevent zoonoses in relevant patients. Earlier studies in Australia revealed that veterinarians are more likely to discuss these risks or strategies, and that lack of vigilance was attributed to GPs' limited awareness and knowledge concerning zoonoses [8,11].

No significant association was found between self-reported higher education on zoonoses during GP training and self-reported confidence in managing zoonoses in practice. This may be because such training often occurs later in their careers, which we did not asses. Developing (online) continuing professional development courses, could improve GPs' knowledge and self-confidence.

A recent study in the Netherlands showed mammalian adaptation of the highly pathogenic avian influenza H5N1, which could represent a risk to human health [14]. However, only a few (15.3 % and 11.8 %) GPs reported to ask about risk factors in patients who experience flu-like symptoms, such as infectious respiratory diseases and fever: this could delay signaling and consequently an adequate response to EIDs but also more ‘well known’ zoonoses, such as psittacosis [15,16]. GPs working in conurbations are less likely to inquire about contact with animals or exposure to nature in patients with neurological disease. This may be because diseases such as Lyme's disease and tick-borne encephalitis are more prevalent in rural areas in the Netherlands, likely making rural GPs more vigilant [[17], [18], [19]]. However, maintaining awareness remains crucial for all GPs, as many patients travel to rural regions for leisure.

GPs who frequently interact with public health authorities, advise patients to consult their veterinarians regarding potential zoonoses and discuss risk and preventive measures, appear to inquire more often about risk factors for zoonoses in specific clinical situations. This could represent a specific group of GPs with an overall higher awareness of zoonoses. Training by infectious disease experts could improve GPs awareness, zoonotic literacy, and multisectoral collaboration, benefiting public health and primary care [20]. One Health simulation exercises could enhance GPs's knowledge of zoonoses and strengthen collaboration with both public health and veterinary professionals.

This is the first study that explored self-reported confidence and anamnesis regarding zoonoses among Dutch GPs, contributing to knowledge on zoonotic literacy. A limitation is the relatively low response rate (7.15 %), common in questionnaire-based surveys without material incentives. However, response bias cannot be excluded, as GPs who are more interested in or concerned about zoonotic diseases may have been overrepresented. This could have resulted in higher reported percentages of GPs who feel confident in diagnosing zoonoses or who routinely ask about zoonotic risk factors. Nevertheless, 47.5 % of respondents indicated that they were not, or slightly, worried about the public health impact of zoonotic diseases, This suggests that the sample was not necessarily overrepresented by highly aware or particularly concerned GPs. Moreover, the sample of about 300 GPs reflected the national age, gender and practice location distribution.

## 5 Conclusions

For an effective response to zoonoses, it is crucial that GPs can recognize these diseases and inquire about risk factors during the anamnesis, as well as understand when and how to report cases to public health authorities. This study showed that only a minority of GPs consider themselves to be adequately informed about their obligations to report notifiable infectious diseases. Moreover, there is significant room for improvement in both awareness of zoonoses and confidence in diagnosing them. GPs would greatly benefit from additional training and information to enhance their ability to diagnose and manage zoonotic signals in primary care. Collaboration between public health and primary care facilitators on this topic should be encouraged. Future research should thus aim to enhance GP education in One Health principles and promote closer collaboration with veterinary and public health sectors.

## Funding

The study was financed by the Ministry of Health, Welfare and Sport through the ZooVer (*Versterking Zoönosen*) research programme.

## CRediT authorship contribution statement

**F. Dusseldorp:** Writing – review & editing, Writing – original draft, Methodology, Formal analysis, Conceptualization. **C.F.J. Vlaanderen:** Writing – review & editing. **S. Feenstra-Gols:** Writing – review & editing. **J.W.B. van der Giessen:** Writing – review & editing, Supervision. **L. Mughini-Gras:** Writing – review & editing, Supervision, Formal analysis.

## Ethics approval and consent to participate

Written informed consent was obtained from the participants of the study. For this study no ethical clearance was needed under the Dutch law, as it did not involve invasive measures. A declaration to this effected was obtained from the Centre for Clinical Expertise at the RIVM (LCI-604, dated 15th February 2023).

## Declaration of competing interest

The authors declare that they have no known competing financial interests or personal relationships that could have appeared to influence the work reported in this paper.

## Data Availability

Data will be made available on request.

## Appendix A Supplementary data

Supplementary material Image 1
