# Supplementary material for: Self-reported knowledge, attitudes and concerns about zoonoses among general practitioners in the Netherlands
Source: One Health. 2025 Jun 27;21:101121. doi: 10.1016/j.onehlt.2025.101121 (PMC12271582; doi:10.1016/j.onehlt.2025.101121)
Supplement: Supplementary file 1 — Supplementary material [file mmc1.docx]

Appendix 1: Questionnaire in Dutch

**Vragenlijst**

Beste collega’s

Wij willen u vriendelijk vragen deze vragenlijst in te vullen over uw ervaringen betreft het signaleren, diagnosticeren en behandelen van zoönosen in de huisartsenpraktijk.

De coronapandemie heeft laten zien dat een zoönose wereldwijd tot ontwrichtende situaties kan leiden. De rijksoverheid heeft daartoe een [Nationaal Actieplan Versterken Zoönosenbeleid](https://www.rijksoverheid.nl/documenten/rapporten/2022/07/06/nationaal-actieplan-versterken-zoonosenbeleid)opgesteld waarin wordt aangegeven hoe de komende 4 jaar het zoönosenbeleid verder wordt versterkt. Een van de pijlers daarvan is zoönosegeletterdheid, zowel bij burgers als professionals. Huisartsen spelen een cruciale rol in het signaleren van bestaande en toekomstige zoönosen bij de mens.

Wij willen u daarom vriendelijk vragen een (pseudoanonieme) vragenlijst in te vullen over uw ervaringen betreft zoönosen in de huisartsenpraktijk. Met de uitkomsten van deze vragenlijst willen we onderzoeken in hoeverre huisartsen zich bekwaam voelen om zoönosen te signaleren, diagnosticeren en behandelen, en in welke mate ze de GGD of andere professionals daarin betrekken. Op de volgende pagina volgt informatie over hoe we uw gegevens vertrouwelijk behandelen en een toestemmingsverklaring.

De vragenlijst zal 5-10 minuten van uw tijd in beslag nemen. Alvast hartelijk dank voor de genomen moeite.

Mocht u nog vragen hebben naar aanleiding van dit onderzoek dan kunt contact opnemen via florien.dusseldorp@rivm.nl.

Met vriendelijke groet,

Florien Dusseldorp
Arts infectieziektebestrijding, AIOS M+G
RIVM, Centrum voor infectieziektebestrijding

**Demografie**

1. Wat is uw geboortejaar?
2. Wat is uw gender?
   1. Vrouw
   2. Man
   3. Transgender
   4. Non-binair
   5. Anders
   6. Wil ik liever niet zeggen
3. Bij welke universiteit heeft u uw geneeskunde opleiding gevolgd?
4. In welk jaar bent u afgestudeerd als huisarts?

------------

1. In welke land bent u afgestudeerd als huisarts?
   a. Nederland
   b. Anders, nl:
2. In welke mate is onderwijs over zoönosen aan bod gekomen in de huisartsenopleiding volgens u?
   1. Ruim voldoende
   2. Voldoende
   3. Onvoldoende
   4. Kwam niet aan bod
3. Heeft u in het buitenland gewerkt en/of gestudeerd als arts resp. in opleiding tot arts?
   Zo ja, waar?
4. In welke provincie is uw (huidige) praktijk gehuisvest*?
   *Voor waarnemers: waar u het meeste werkt
5. In wat voor omgeving staat uw (huidige) praktijk?
   1. Grote stad (>100.000 inwoners)
   2. Kleine stad (<100.000 inwoners)
   3. Dorp of platteland

*Voor waarnemers: waar u het meeste werkt

1. Hoe zelfverzekerd voelt u zich als huisarts in het algemeen?
   1. Heel onzeker
   2. Onzeker
   3. Neutraal
   4. Zeker
   5. Heel zeker

**Infectieziekten en meldplicht**

1. Hoe zelfverzekerd voelt u zich in het diagnosticeren en managen^[[1]](#footnote-1)^ van een infectieziekte bij een patiënt?
   1. Heel onzeker
   2. Onzeker
   3. Neutraal
   4. Zeker
   5. Heel zeker
2. Hoe goed bent u op de hoogte van je plicht als arts om bepaalde infectieziekten en/of situaties te melden aan de GGD in het belang van de volksgezondheid?
   1. Helemaal niet
   2. Een beetje
   3. Redelijk
   4. Goed
   5. Heel goed
3. Hoe vaak heeft u contact opgenomen met de GGD om een meldingsplichtige ziekte door te geven (laat daarbij COVID-19 en Mpox buiten beschouwing)?
   1. Nooit
   2. Sporadisch
   3. Regelmatig
   4. Vaak
   5. Heel vaak
4. Heeft u wel eens contact opgenomen met het team infectieziekten van de GGD om te overleggen over een infectieziekte?
   1. Ja
   2. Nee
   3. Weet ik niet

**Zoönosen**

1. Hoe bezorgd bent u over ontwikkelingen op het gebied van zoönosen voor de volksgezondheid?
   1. Niet bezorgd
   2. Enigszins bezorgd
   3. Matig bezorgd
   4. Erg bezorgd
   5. Heel erg bezorgd
2. Hoe zelfverzekerd voelt u zich in het diagnosticeren en managen^1^ van een zoönose bij een patiënt?
   1. Heel onzeker
   2. Onzeker
   3. Neutraal
   4. Zeker
   5. Heel zeker
3. Hoe vaak diagnosticeert u een zoönose bij een patiënt per jaar?
   1. Minder dan 1x per jaar
   2. 1-5x per jaar
   3. >5x per jaar
4. Welke zoönosen heeft u wel eens gediagnosticeerd?
   1. Geen een
   2. De volgende zoönosen:
5. Heeft u wel eens een patiënt aanbevolen de dierenarts te consulteren om zijn/haar huisdieren mee te laten behandelen bij een gediagnosticeerde zoönose?
   1. Ja
   2. Nee
6. Heeft u wel eens een patiënt doorverwezen gekregen vanuit de dierenarts vanwege blootstelling aan of verdenking op een zoönose?
   1. Ja
   2. Nee
7. Hoe vaak bespreekt u de risico’s en preventie van zoönosen bij (relevante) patiënten?
   1. Nooit
   2. Zelden
   3. Soms
   4. Vaak
   5. Altijd
8. Vraagt u in de volgende (klinische) situaties naar (de aard van) contact met dieren of mogelijke blootstelling aan dieren in de natuur?
   1. Ziekte na een vakantie/reis: (vrijwel) altijd/meestal/soms/(vrijwel) nooit
   2. Ziekte in de zwangerschap: (vrijwel) altijd/meestal/soms/(vrijwel) nooit
   3. Ziekte bij mensen met een beroepsmatig risico (boeren/groenwerkers bv.): (vrijwel) altijd/meestal/soms/(vrijwel) nooit
   4. Respiratoire klachten: (vrijwel) altijd/meestal/soms/(vrijwel) nooit
   5. Neurologische klachten: (vrijwel) altijd/meestal/soms/(vrijwel) nooit
   6. Huidinfecties: (vrijwel) altijd/meestal/soms/(vrijwel) nooit
   7. Koorts: (vrijwel) altijd/meestal/soms/(vrijwel) nooit
9. Hoe zou u het liefst informatie/kennis willen ontvangen over zoonosen?
   1. In de opleiding
   2. Middels nascholing
   3. Via wetenschappelijke artikelen/tijdschriften
   4. Via GGD’en
   5. Anders, nml:

1. Behandelen, verwijzen, adviseren of overig wat passend is in de situatie van de betreffende patiënt [↑](#footnote-ref-1)
